# Using the Sensible Heat Flux Eddy Covariance-Based Exchange Coefficient to Calculate Latent Heat Flux from Moisture Mean Gradients Over Snow

**DOI:** 10.1007/s10546-024-00864-y

**Published:** 2024-05-04

**Authors:** Sergi González-Herrero, Armin Sigmund, Michael Haugeneder, Océane Hames, Hendrik Huwald, Joel Fiddes, Michael Lehning

**Affiliations:** 1 WSL Institute for Snow and Avalanche Research (SLF), Davos, Switzerland; 2 Environmental Engineering Institute, Laboratory of Cryospheric Sciences, Ecole Polytechnique Fédérale de Lausanne (EPFL), Valais/Wallis, Sion, Switzerland

**Keywords:** Eddy-covariance, Monin–Obukhov similarity theory, Latent heat flux, Snow, Boundary layer, Bowen ratio

## Abstract

In absence of the high-frequency measurements of wind components, sonic temperature and water vapour required by the eddy covariance (EC) method, Monin–Obukhov similarity theory (MOST) is often used to calculate heat fluxes. However, MOST requires assumptions of stability corrections and roughness lengths. In most environments and weather situations, roughness length and stability corrections have high uncertainty. Here, we revisit the modified Bowen-ratio method, which we call C-method, to calculate the latent heat flux over snow. In the absence of high-frequency water vapour measurements, we use sonic anemometer data, which have become much more standard. This method uses the exchange coefficient for sensible heat flux to estimate latent-heat flux. Theory predicts the two exchange coefficients to be equal and the method avoids assuming roughness lengths and stability corrections. We apply this method to two datasets from high mountain (Alps) and polar (Antarctica) environments and compare it with MOST and the three-layer model (3LM). We show that roughness length has a great impact on heat fluxes calculated using MOST and that different calculation methods over snow lead to very different results. Instead, the 3LM leads to good results, in part due to the fact that it avoids roughness length assumptions to calculate heat fluxes. The C-method presented performs overall better or comparable to established MOST with different stability corrections and provides results comparable to the direct EC method. An application of this method is provided for a new station installed in the Pamir mountains.

**Supplementary Information:**

The online version contains supplementary material available at 10.1007/s10546-024-00864-y.

## Introduction

Despite its relevant role on the surface mass balance, the latent heat flux above snow and ice is one of the most poorly described processes in high mountain and polar regions. This process depends on the turbulent exchange of moisture in the lower atmosphere. Prandtl (1925), addressed the problem of wall-bounded turbulent flows and associated exchange of momentum and scalars. Suggesting a mixing length argument, he introduced the now-famous logarithmic law of the wall, which analytically predicts the velocity profile of the turbulent boundary layer for the case of a constant momentum flux, which requires the assumption of horizontal homogeneity and stationarity. The fundamental idea, that a constant flux must be present near the surface for steady and homogeneous conditions, forms the basis of the formulation of so-called bulk transfer models for momentum and scalars alike, which predict exchange with the surface as a function of differences in mean variables between two levels in the turbulent layer close to the surface. In practice, the difference is often taken between the surface and some (arbitrary) height in the surface layer, the lowest part of the atmospheric boundary layer. The height needs to be within the constant flux layer. Since atmospheric (weather and climate) but also snow, soil, hydrologic, fluid engineering and vegetation models typically solve partial differential equations on a numerical grid, those bulk formulae are the method of choice for quantifying exchange across the flow–surface interface.

The difference between the surface and the lowest grid point in the flow is taken to provide forcing of exchange in the form:

1 $${Q}_{x}\sim \Delta x \Delta v,$$

where x is any scalar or vector quantity to be transported, v is the mean velocity of the turbulent flow and Q is the (vertical) flux/exchange. This double-proportionality, modified by atmospheric stability effects, has proven to be fulfilled in many situations and even to be a practical approximation in cases where the assumptions of horizontal homogeneity and stationarity are not fulfilled (Nadeau et al. 2013; Grachev et al. 2016; Lehner and Rotach 2018; Sfyri et al. 2018). The factor of proportionality can be theoretically estimated by e.g. Prandtl mixing length arguments or more generally through the Monin–Obukhov similarity theory (MOST) (Monin and Obukhov 1954). The surface exchange model, often called law of the wall, works typically best, if the lowest grid point in the atmosphere is located close to the surface but within the fully turbulent flow region, because then the complication of overlapping internal boundary layers is avoided (Haugeneder et al. 2023). Furthermore, in cases of drifting and blowing snow, the presence of snow particles leads to the observation that bulk transfer severely underestimates local fluxes as recently demonstrated by Sigmund et al. (2022).

In-situ observations are used to validate surface moisture exchange, and hence surface sublimation or deposition in models (e.g., King and Anderson 1994; Box and Steffen 2001; Stössel et al. 2010; Reba et al. 2012). However, the measurement of turbulent moisture exchange is a challenging task. Direct measurements rely on the eddy-covariance (EC) method that requires high-frequency measurements of both wind and moisture. The use of an infrared gas analyzer as part of an EC system adds sources of uncertainty to the measurements such as time lag and density fluctuations; these problems are mostly solved by an exhaustive postprocessing (Massman and Lee 2002; Burba 2022). However, gas analysers, especially open path systems, and other fast-response hygrometers are still more sensitive to obstacles in the air, riming, and icing than sonic anemometers, and also need to be well calibrated for temperature variations. Integrated infrared gas analyzers are expensive and not reliable enough for long-term unsupervised deployment and the logistic difficulties associated with regular maintenance prevent the development of a widespread network with EC moisture flux measurements. Although this can be solved in the future with new developments of cheap EC sensors with fast-response humidity measurements such as the recently developed LI-710 (LI-COR, Lincoln, USA), they are not yet widely deployed and their performance in snow environments still has to be evaluated. When high-frequency measurements of water vapour are not available, moisture flux is estimated using the MOST parametrization, which only requires low-frequency measurements with cheaper instruments. However, this method relies on the assumption of a horizontally homogeneous and flat surface, which is not fulfilled in many snow-covered regions that are located in complex terrain. Furthermore, a proper quantification of atmospheric stability corrections and the roughness length, which have a significant effect on the quantification of the flux exchange (Optis et al. 2016; Schlögl et al. 2017), is needed. Due to these limitations, the direct EC method for measuring fluxes is generally more reliable than MOST parametrizations. Specifically, in snow environments, MOST parametrizations are still applicable in flat polar terrains after including universal functions for stable stratification over ice and snow (Grachev et al. 2013; Gryanik et al. 2020, 2021; Gryanik and Lüpkes 2023). However, since MOST is only valid for the turbulent surface layer, a high temperature gradient in the molecular and buffer layers can develop above very smooth ice and snow surfaces and might lead to errors in MOST when using the surface temperature and moisture (Sodemann and Foken 2005). Furthermore, MOST might also face limitations in mountainous terrain due to the complex orography introducing turbulence anisotropy (Stiperski and Calaf 2023).

In recent years, 3D high-frequency sonic anemometers have become increasingly integral instruments in meteorological stations. Consequently, some sites only offer high-frequency measurements of wind and virtual temperature, allowing sensible heat flux (*Q*_*s*_) but not latent heat flux (*Q*_*l*_) to be estimated using EC methods. In these cases, it is difficult to compare *Q*_*s*_ and* Q*_*l*_, unless MOST parameterizations are used for the computation of both fluxes. However, this issue can be effectively resolved by employing the modified Bowen-ratio method or the bandpass EC method (Businger 1986; Watanabe et al. 2000). The former requires measurements of vertical differences of humidity and temperature while the latter uses a slow-response hygrometer and estimates high-frequency components of the flux by means of co-spectral similarity. The objective of this paper is to revisit the modified Bowen-ratio theory without explicitly going through the Bowen-ratio calculation and thus, to compute *Q*_*l*_ using only high-frequency wind and temperature measurements avoiding stability and roughness approximations. The modified Bowen-ratio method has been extensively applied for different surfaces (e.g. Weber 2006; Mauder et al. 2007; Dunn et al. 2009), including some snow-covered surfaces (van As et al. 2005; Amschwand et al. 2023). However, it is important to note that this method has not undergone specific evaluation in snowy environments—a surface characterized by unique challenges for estimating turbulent heat exchange, such as small roughness elements and stable stratification—which is the primary focus of our study. We compare this methodology with in-situ EC measurements of *Q*_*l*_ in high mountain (Alps) and polar (Antarctica) environments. We show its performance with respect to other methods of calculating heat fluxes and discuss the utility for snow covered surfaces. Finally, we show an application in the Pamir mountain range, where long-term year-round micrometeorological measurements with only sonic anemometers are being conducted. With these measurements we estimate the sublimation without high-frequency moisture data.

## Data and Methods

### Theoretical Background

In this section we revisit the modified Bowen ratio method (Businger 1986; Liu and Foken 2001) focusing on the direct utilization of the exchange coefficient for the computation of the latent heat flux. Vertical turbulent fluxes can be calculated by the EC method using high-frequency measurements of the covariance components. Sensible and latent heat fluxes are calculated by:

2 $${Q}_{s}={\rho }_{air}{c}_{p} \overline{w^{\prime}\theta ^{\prime}},$$

3 $${Q}_{l}={\rho }_{air}{L}_{s} \overline{w^{\prime}q^{\prime}},$$

where ${\rho }_{air}$ (kg m^−3^) is the density of the air, ${c}_{p}$ (J kg^−1^ K^−1^) is the air heat capacity, ${L}_{s}$ (J kg^−1^) is the latent heat of sublimation and $\overline{w^{\prime}\theta ^{\prime}}$ and $\overline{w^{\prime}q^{\prime}}$ are the averaged covariances between the vertical velocity component w (m s^−1^) and the potential temperature $\theta$ (K) and specific humidity $q$ (kg kg^−1^), respectively.

When the high-frequency measurements are not available, the sensible and latent heat fluxes can be estimated using the Monin–Obukhov similarity theory (MOST). Using this method, the sensible and latent heat fluxes are expressed as:

4 $${Q}_{s}=- {C}_{s} \overline{{\text{U}}} {\rho  }_{air} {c}_{p} ({\theta }_{z}-{\theta }_{0}),$$

5 $${Q}_{l}=-{C}_{l} \overline{{\text{U}}} {\rho  }_{air} {L}_{s} ({q}_{z}-{q}_{0}),$$

where ${\theta }_{z}$ and ${\theta }_{0}$ (K) are the average potential temperatures at height z (m) and the surface, and ${q}_{z}$ and ${q}_{0}$ (kg kg^−1^) are the average values of specific humidity at the same levels, while $\overline{{\text{U}} }$ is the average wind speed at height z. Finally, ${C}_{s}$ and ${C}_{l}$ are the dimensionless turbulent exchange coefficients for sensible and latent heat, respectively. Both coefficients depend on stability and height and are given by:

6 $${C}_{s}=\frac{{\left(\frac{\kappa }{{Pr}_{t}}\right)}^{2}}{\left[ln \left(\frac{z}{{z}_{0T}}\right) -{\psi }_{T}(\zeta )\right]\left[ln \left(\frac{z}{{z}_{0M}}\right) -{\psi }_{m}(\zeta )\right]},$$

7 $${C}_{l}=\frac{{\left(\frac{\kappa }{{Sc}_{t}}\right)}^{2}}{\left[ln \left(\frac{z}{{z}_{0q}}\right) -{\psi }_{q}(\zeta )\right]\left[ln \left(\frac{z}{{z}_{0M}}\right) -{\psi }_{m}(\zeta )\right]},$$

where *κ* = 0.40 is the von Kármán constant (Högström 1988), *Pr*_*t*_ and *Sc*_*t*_ are the turbulent Prandtl and Schmidt numbers, $\zeta$ is the dimensionless Obukhov stability parameter, ${z}_{0T}$, ${z}_{0q}$, and ${z}_{0M}$ are the roughness lengths for temperature, humidity, and momentum, respectively, and ${\psi }_{T}$, ${\psi }_{q}$ and ${\psi }_{m}$ are the integrated stability correction functions for sensible heat, latent heat and momentum, respectively. Several empirical studies suggest that both ${C}_{s}$ and ${C}_{l}$ have an order of magnitude of 10^–3^ over snow and the ratio ${C}_{s}$/${C}_{l}$ is in the interval 0.2–1.0 (Hicks and Martin 1972; Thorpe et al. 1973; Kondo and Yamazawa 1986; Andreas 1987). While these findings generally suggest a slightly higher value for C_l_ compared to C_s_, uncertainties arise from methodological inaccuracies and the dependency on stable stratification and height variations, making it challenging to pinpoint an exact numerical relationship (Li 2019; Foken and Mauder 2024). A general convention, especially among modelers, is to use ${C}_{l}={C}_{s}$ (e.g. Martin and Lejeune 1998; Vionnet et al. 2012). The latter assumption is consistent with the MOST theory that assumes stationarity and homogeneity in the layer above the ground. Therefore, scalar transport is achieved by turbulent eddies, which transport heat and moisture in exactly the same way.

Using high-frequency measurements of wind and temperature, which are easier to obtain than for water vapour, *C*_*s*_ can also be obtained by combining Eqs. 2 and 4:

8 $${C}_{s}=\frac{{Q}_{s}}{ {\rho }_{air}{c}_{p}\overline{{\text{U}} } \left({\theta }_{z}-{\theta }_{0}\right)}=\frac{- \overline{w^{\prime}\theta ^{\prime}}}{ \overline{{\text{U}} } \left({\theta }_{z}-{\theta }_{0}\right)}.$$

Considering *C*_*s*_ = *C*_*l*_, *Q*_*l*_ can be calculated using Eq. 5, avoiding the approximation of the stability corrections in Eq. 7:

9 $${Q}_{l}={C}_{s} \overline{{\text{U}}} {\rho  }_{air} {L}_{s} \left({q}_{z}-{q}_{0}\right)=\frac{ \overline{{w }^{\prime}{\theta }^{\prime}}}{ \overline{{\text{U}} } \left({\theta }_{z}-{\theta }_{0}\right)} \overline{{\text{U}}} {\rho  }_{air} {L}_{s} \left({q}_{z}-{q}_{0}\right)=\frac{{L}_{s}}{{c}_{p}}\frac{\left({q}_{z}-{q}_{0}\right)}{\left({\theta }_{z}-{\theta }_{0}\right)}{Q}_{s}.$$

In the rest of the paper we will refer to this derivation as exchange coefficient method or C-method for abbreviation, since it directly uses the exchange coefficient to obtain the latent heat flux. Our derivation connects to the modified Bowen ratio method when the Bowen ratio (Bo), defined as the ratio of sensible and latent heat fluxes, is considered:

10 $$Bo=\frac{{Q}_{s}}{{Q}_{l}}=\frac{{c}_{p}}{{L}_{s}}\frac{\left({\theta }_{z}-{\theta }_{0}\right)}{\left({q}_{z}-{q}_{0}\right)}.$$

Using Eqs. 10, 9 can be rewritten as

11 $${Q}_{l}=\frac{{Q}_{s}}{Bo}.$$

**Fig. 1:**
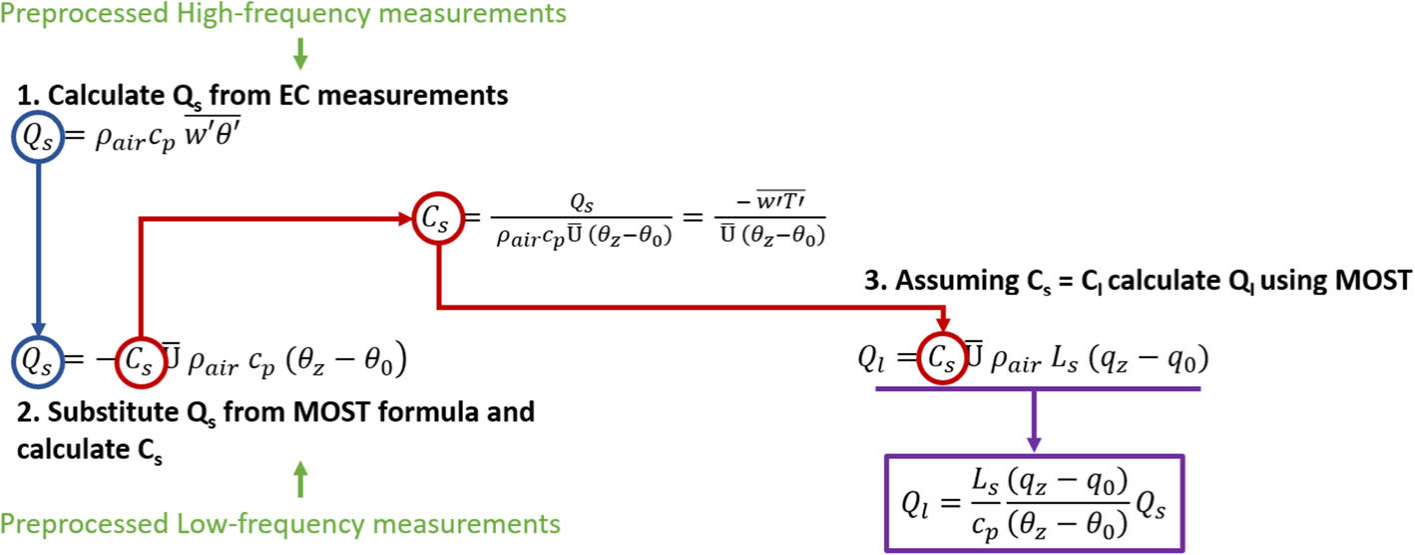
Scheme of the derivation of the C-method

This simple approach is sketched in Fig. 1, and the main advantage is that it does not depend on the choice of the stability approximation and the roughness length. We want to avoid to use the term “Bowen Ratio” when referring to the method, because Bowen Ratio stands for a flux similarity assumption, while the C-method only uses the theoretical result of equal exchange coefficients.

### Datasets

**Fig. 2:**
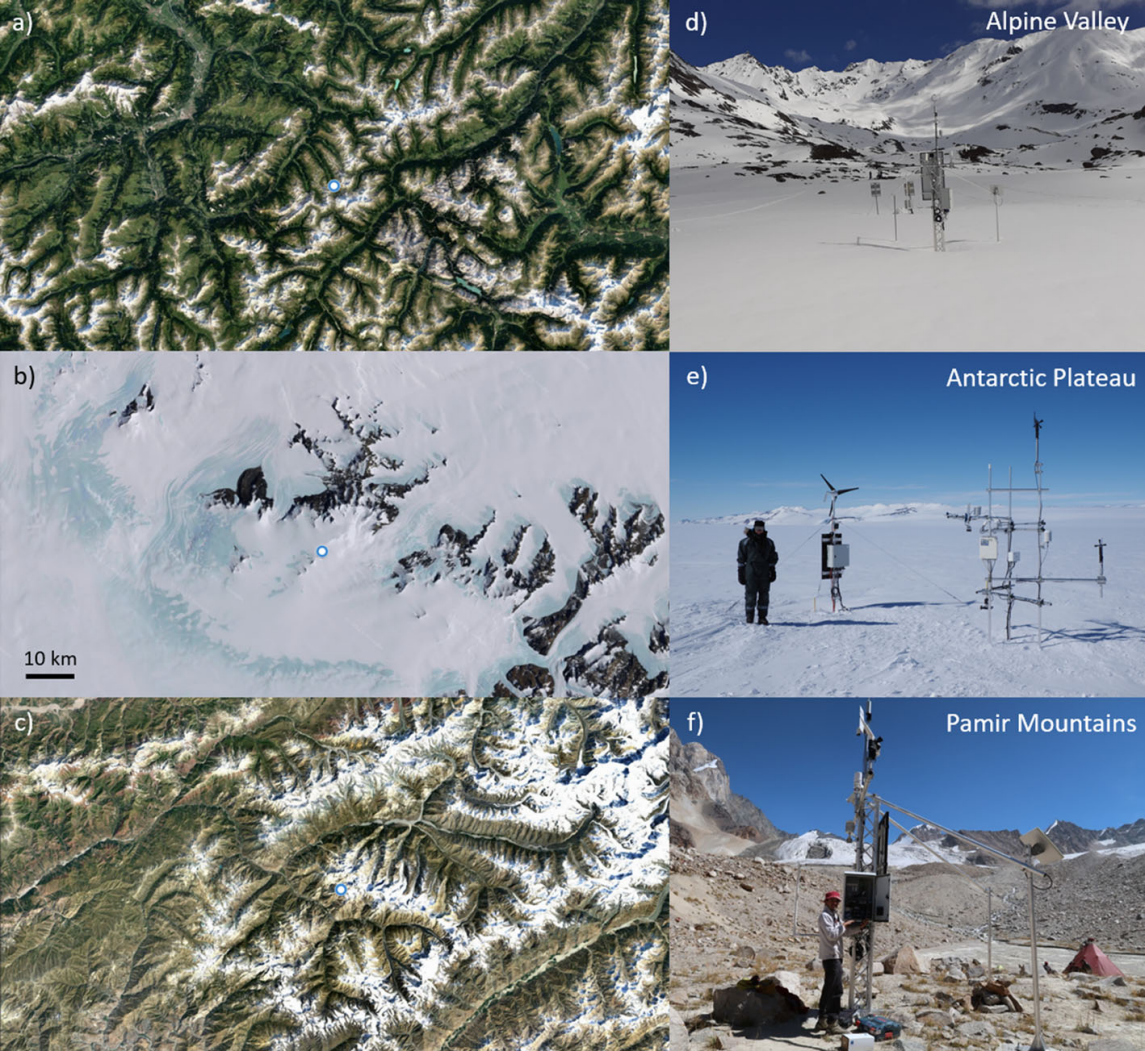
Location (**a**–**c**) and picture (**d**–**f**) of the stations used in this research in the Alpine Valley (**a**, **d**), the Antarctic plateau (**b**, **e**), and Pamir mountains (**c**, **f**). Notice that the picture in f is without snow but the measurements used are during the snow season. The scale in (**b**) is the same in all maps

For the validation of the methodology we used a dataset from automatic EC sensors to calculate sensible and latent heat fluxes in two different snow environments: An Alpine valley and the Antarctic plateau. Then, we applied the methodology to a station in the Pamir mountains, which does not have a high-frequency humidity sensor. Stations and their surroundings are shown in Fig. 2, and the instruments used for calculating fluxes and their initial heights are shown in Table 1.

**Table 1:** Variables and instruments used for EC and MOST measurements and initial heights for the SNOWPACK simulations

Variable	Instrument	Model	Height (m)	Time interval	Method
*Alpine Valley*					
High-frequency wind components, sonic temperature	Ultrasonic anemometer	Campbell Scientific CSAT3 (from IRGASON)	1	0.05 s	EC
Water-vapour molar density, air pressure	Infrared gas analyzer	Campbell Scientific IRGASON	1	0.05 s	EC
Wind speed and direction	Propeller anemometer	Young Wind Monitor 05103–5	3.1	10 min	MOST
Air temperature, relative humidity	Temperature / humidity prove	Campbell HygroVUE10	3.7	10 min	MOST
Surface temperature	Infrared radiometer	Campbell SI-111	2.2	10 min	MOST
Snow height	Laser snow depth sensor	Lufft SHM31	2.7	10 min	MOST
*Antarctic Plateau*					
High-frequency wind components, sonic temperature	Ultrasonic anemometer	Campbell Scientific CSAT3	2.1	0.1 s	EC
Water-vapour molar density, air pressure	Infrared gas analyzer	LICOR LI-7500	2.1	0.1 s	EC
Wind speed and direction	Propeller anemometer	Young Wind Monitor 05103	1.67	10 min	MOST
Air temperature, relative humidity	Temperature / humidity prove	Campbell HygroVUE10	1.8	10 min	MOST
Surface temperature	Infrared radiometer	Campbell SI-111	2.2	10 min	MOST
Snow height	Ultrasonic snow sensor	SR50A	1.9	10 min	MOST
*Pamir Mountains*					
High-frequency wind components, sonic temperature	Ultrasonic anemometer	Campbell Scientific CSAT3	4.4	0.05 s	EC
Wind speed and direction	Propeller anemometer	Young Wind Monitor 05103	3.1	10 min	MOST
Air temperature, relative humidity	Temperature / humidity prove	Campbell HygroVUE10	3.7	10 min	MOST
Surface temperature	Infrared radiometer	Campbell SI-111	2.2	10 min	MOST
Snow height	Ultrasonic snow sensor	Lufft SHM31	2.7	10 min	MOST

#### Alpine Valley

Measurements at the Alpine valley were obtained from Dischma valley (46.72004° N, 9.92287° E at 2020 m a.s.l.) near Davos in the Swiss Alps from 21st to 25th May 2021 prior to the start of the melting of the snowpack and the presence of the first snow-free patches. The station was located in a flat region at the end of the valley surrounded by steep slopes including a small glacier in the south. During the measurement campaign, the vegetation was completely covered by snow. Therefore, the flow was mainly influenced by synoptic forcing and katabatic down-valley winds. The station measured high-frequency (20 Hz) wind velocity components, sonic temperature and water-vapour molar density using an IRGASON (Campbell Scientific, Logan, USA). We also used low-frequency (10 min) measurements of wind speed and direction, surface and near-surface air temperature, relative humidity, and snow height.

#### Antarctic Plateau

The dataset over the Antarctic plateau was measured at the southern edge of the Sør Rondane mountains in Dronning Maud Land (72.25150° S, 23.26922° E at 2313 m a.s.l.) from 18th December 2021 to 14th March 2022. The closest research base is Princess Elisabeth Station at a horizontal distance of approximately 34 km. The immediate surroundings of the measurement station are nearly flat and permanently covered by snow while a small nunatak and a mountain range are located approximately 1 km south and 5 km northeast of the station, respectively. The terrain is slightly sloping towards the northeast with an angle of approximately 3°. Most of the time, the site experiences easterly winds with an unobstructed fetch of at least 14 km. The station measured the same components as the one in the Alpine valley (Table 1) but with 1-min resolution for the low-frequency measurements and 10 Hz for the high-frequency measurements using CSAT3 ultrasonic anemometer (Campbell Scientific, Logan, USA) for wind and sonic temperature and a LI-7500 (LI-COR, Lincoln, USA) for water-vapour molar density.

#### Pamir Mountains

The methodology was applied to measurements obtained from a station at Sangvor (38.74287° N, 71.35392° E at 3909 m a.s.l.), located in the Western Pamir Mountains, a region of Central Asia exposed to the synoptic weather systems and relatively humid. The station is located on a glacial forefield plain in a valley oriented from SE to NE approximately 1 km away of the Nissai Glacier moraine. It is surrounded by steep mountains, specially to the southwest, that force the katabatic down valley winds from the southeast. The dataset spans the entire 2021–2022 snow season, which lasted from 21 October 2021 to 01 June 2022. Measurements were similar to those in the Alpine Valley station, but due to power constraints, only a CSAT3 with high-frequency measurements (20 Hz) of wind velocity components and sonic temperature was installed, and not water-vapour molar density. However, 48% of the Q_s_ data was discarded due to their quality, especially because they did not pass the steady state test.

### Processing

#### Eddy-Covariance Dataset

The EC data was processed using the methodology described by Sigmund et al. (2022; see their Appendix A), which has two main steps. First, an initial pre-processing, in which artefacts are removed based on sensor diagnostics and plausibility limits, followed by a bias correction for vapour density and a spike removal algorithm. Second, a processing using the EddyPro® software (LI-COR 2021) with a Reynolds averaging of 20 min, time lag, density fluctuations and spectral loss corrections and double coordinate rotation. We also incorporated the Burba et al. (2008) correction with default parameters for data from the LI-7500 sensor to address the sensor heating effect. In contrast to Sigmund et al. (2022), the flux correction for the difference between sonic temperature and air temperature (van Dijk et al. 2004), also known as the SND correction (Schotanus et al. 1983), is not implemented, since this correction requires the latent heat flux. Therefore, measurements without this correction represent the kinematic flux of virtual potential temperature (i.e. the buoyancy flux divided by the buoyancy parameter g/T_v_) rather than the sensible heat flux. The error of using the kinematic flux of virtual potential temperature instead of the sensible heat flux can be considered very small in snow-dominated environments as absolute humidity is low. We validated this approximation by comparing the kinematic flux of virtual potential temperature ($\overline{w^{\prime}{\theta }_{s}^{\prime}}$) and sensible heat flux ($\overline{w^{\prime}\theta ^{\prime}}$), using the correction of van Dijk et al (2004), which does not depend on the momentum flux in contrast to the correction of Schotanus et al. (1983). The median error committed by using buoyancy fluxes without SND correction is 2.2% (with an interquartile range of 0.8–4.8%) in the Alpine valley and 1.5% (with an interquartile range of 1.1–2.0%) on the Antarctic Plateau. This error is small compared to other uncertainties. We adopt this assumption throughout the rest of this manuscript. Finally, we included a quality-control flag similar to Sigmund et al. (2022) as a combined result of the steady state, well-developed turbulence and NaN tests, and accounting for the interdependence of *Q*_*s*_ and *Q*_*l*_ (i.e. increasing + 1 if the quality of the other flux is bad following Mauder et al. (2013). For the results presented, low quality values (quality flag equal to 2) were discarded.

Since occasional decoupling occurs below the instruments over the snow surface, counter-gradient fluxes might appear (Foken 2023). Under these conditions, the similarity theory does not apply and therefore also our method, which relies on it, fails. To avoid these conditions, negative values of Cs were discarded, and latent heat fluxes were not calculated using the C-method.

#### MOST Calculations

The MOST surface turbulent heat fluxes were calculated using the physics-based snow-surface model SNOWPACK (Lehning et al. 2002). To force the model, we used the meteorological data of near-surface air temperature, humidity and wind at heights specified in Table 1. Measured surface temperature was employed as a proxy for the lower level in the MOST formulation assuming water vapour saturation over the surface, which is common practice for snow and ice surfaces (Andreas 2002). This approach assumes that the main source of uncertainty stems from the roughness lengths (Mahrt and Vickers 2004). We used a 1-min time step (with data interpolation) and averaged the outputs every 20 min to compare with calculated EC fluxes. The roughness lengths of temperature and humidity for each site were set to the same value for momentum and were estimated using data from the EC measurements with near neutral stratification (− 0.1 < *z/L* < 0.1) to avoid uncertainties due to stability effects and the formula of the logarithmic wind profile, so that:

12 $${z}_{0M}=z \cdot  exp(-\kappa \frac{\overline{{\text{U}}}}{{u }_{*}}),$$

where ${u}_{*}$ is the friction velocity and $\overline{{\text{U}} }$ is the wind velocity at the height *z*. Furthermore, we removed the values that did not pass the ${\sigma }_{u}/{u}_{*}$ test to limit the effect of inhomogeneous terrain (Foken and Leclerc 2004). We chose the roughness length as the median of all values in neutral stratification. We obtained 7 × 10^–3^ m (*N* = 73) for the Alpine Valley, 2 × 10^–4^ m (*N* = 171) for the Antarctic Plateau and 0.17 m (N = 3461) for the Pamir mountains.

Low roughness lengths over snow can be susceptible to significant errors due to minor inaccuracies in wind speed measurements (Foken and Mauder 2024). Therefore, we tested the alternative derivation of roughness length proposed by Panofsky (1984) (from now on P84), who determined the roughness length based on integral turbulence characteristics under neutral conditions:

13 $$ z_{{0M}}  = z/exp\left( {1.25\kappa \frac{{\overline{{\text{U}}} }}{{\sigma _{w} }}} \right). $$

This approach yielded very different roughness lengths of 3 × 10^–4^ m for the Alpine Valley, 7 × 10^–5^ m for the Antarctic Plateau and 0.05 m for the Pamir mountains.

Main results are compared with MOST, computed using the estimated roughness lengths in Eqs. (12) and (13). The Holtslag stability correction was selected as reference because it is one of the most widely used corrections for different models and reanalysis data such as ECMWF/ERA5 (ECMWF 2016) or CRYOWRF (Sharma et al. 2023) and has proven to be successful over snow surfaces (Schlögl et al. 2017).

For each site we performed a sensitivity analysis with respect to the choice of roughness length and stability correction. We tested five different roughness lengths (1/3, 2/3, 1, 4/3 and 5/3 of the estimated roughness length in Eq. (12)) with the Holtslag stability correction, and seven other stability corrections with the estimated roughness length in Eq. (12). The stability corrections tested were:

1. Simplified **Richardson** number correction
2. Assuming **neutral** stratification
3. A simple **Log-linear** model (Webb 1970) with an empirical parameter β = 5 (Marks and Dozier 1992; Schlögl et al. 2017)
4. **Holtslag** correction with linear and exponential terms (Holtslag and de Bruin 1988)
5. **Stearns** correction with logarithmic and inverse tangent terms (Stearns and Weidner 2011)
6. **Schlögl univariate** parametrization assuming a conventional linear dependence of the stability parameter (Schlögl et al. 2017)
7. **Schlögl multivariate** parametrization with offset assuming a dependence on buoyancy and shear terms in a first order statistical model (Schlögl et al. 2017)

These atmospheric stability corrections require a standard iterative method, except when simply assuming neutral stratification. They cover stable conditions while rare cases of unstable conditions are treated using the correction of Paulson (1970) for momentum and that of Stearns and Weidner (2011) for latent and sensible heat.

#### Three-Layer Model

We used a hydrodynamic 3-layer model (3LM) to identify a possible decoupling between the lowest atmospheric layers (where the molecular exchange processes dominate) and the turbulent layer due to either significant long-wave radiation cooling or a katabatic flow over a cold air pool (Foken 1984; Sodemann and Foken 2005). Strong deviations between the 3LM and EC results indicate decoupling. The 3LM integrates the diffusion coefficients over the molecular, buffer and turbulent layers. In this way, the sensible heat flux can be determined using the profile coefficient $\Gamma$, so:

14 $${Q}_{s}=-\Gamma {\rho }_{air} {c}_{p} ({\theta }_{z}-{\theta }_{0}),$$

with:

15 $$\Gamma ={\left({\int }_{0}^{z}\frac{dz}{{K}_{T}+{\upsilon }_{Tt}+{\upsilon }_{T}}\right)}^{-1},$$

where ${K}_{T}$ is the turbulent exchange coefficient for heat in the turbulent layer, ${\upsilon }_{Tt}$ is the molecular-turbulent exchange coefficient in the buffer layer, and ${\upsilon }_{T}$ is the molecular exchange coefficient in the molecular layer.

The integration of the profile coefficient by Lüers and Bareiss (2010) results in:

16 $$\Gamma =\frac{\kappa {u}_{*}}{\frac{{\delta }_{T}{u}_{*}}{\upsilon }\kappa Pr+4\kappa +ln\frac{{u}_{*}z}{30\upsilon }},$$

where $\upsilon$ is the kinematic viscosity of the air and ${\delta }_{T}$ is the dimensionless temperature difference in the buffer layer, with:

17 $$\frac{{\delta }_{T}{u}_{*}}{\upsilon }=\left\{\begin{array}{c}6, {u}_{*}\le 0.23 m{s}^{-1}\\ 12, {u}_{*}>0.23 m{s}^{-1}\end{array}\right. .$$

This profile coefficient calculated in Eq. (16) is then applied to Eq. (14) to derive Q_s_, which is used to compute Q_l_ using the C-method. Finally, rearranging Eq. (14) with the measured Q_s_ by EC method it can be also obtained an estimate of the modelled surface temperature ${\theta }_{0}$.

## Results

### Overview of the Selected Cases

**Fig. 3:**
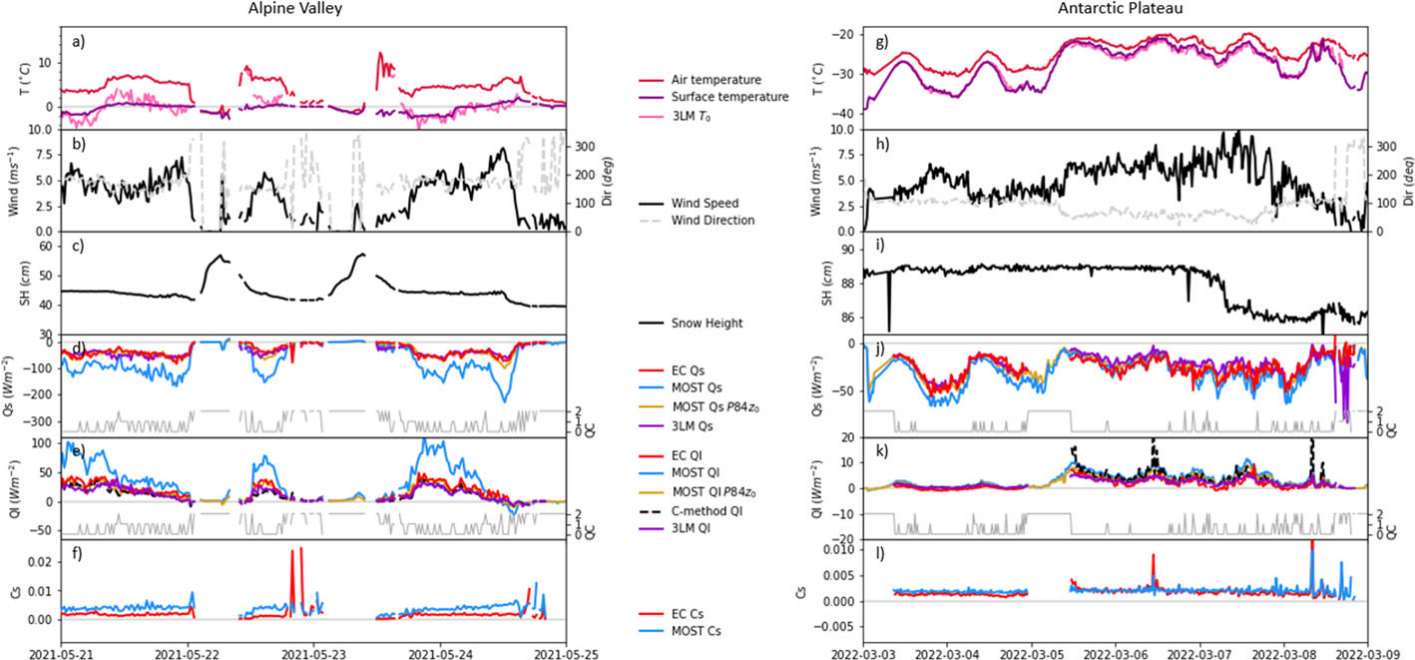
Timeseries of near-surface air temperature and surface temperature (**a**, **g**), wind speed and direction (**b**, **h**), snow height (**c**, **i**), sensible heat flux (**d**, **j**), latent heat flux (**e**, **k**) and exchange coefficient (**f**, **l**) in the Alpine Valley (**a**–**f**) and the Antarctic Plateau (**g**–**l**). Sensible and latent heat fluxes are shown for different methods of computation with Eddy Covariance measurements (EC), Monin–Obukhov similarity theory (MOST), the three-layer model (3LM) and the C-method. MOST calculations shown in this figure use Holtslag stability correction and the estimated roughness length using the logarithmic wind profile in neutral conditions and the P84 formulation (see methodology). Gray lines in **d**, **e**, **j**, **k** show the quality control values that indicate “0” for best quality fluxes, “1” for fluxes suitable for general analysis such as annual budgets and “2” for fluxes that should be discarded from the results dataset (Mauder et al. 2013). Values are represented as 20-min averages and dates are shown in local time: UTC+ 1 h for Alpine Valley and UTC+ 3 h for Antarctic Plateau

Figure 3 shows the time evolution of the two periods used to test the C-method presented in Sect. 2. The alpine valley event was recorded in late spring, just days before the onset of an extensive snowmelt that led to the first snow-free patches in the area (Haugeneder et al. 2023). The surface temperature was around 0 °C throughout the period and the near-surface air temperature was between 0 and 10 °C for most of the day with two calm nights where near-surface air temperature dropped until it reached surface temperature (Fig. 3a). These two nights were characterized by light snowfalls that caused an increase in the snow cover of about 15 cm. The new snow, which was lighter than the previous wet, compressed snowpack, compacted and melted rapidly in the morning (Fig. 3c). When near-surface air temperatures were positive, *Q*_*s*_ values around − 50 Wm^−2^ were measured using EC (Fig. 3d). However, the estimates of *Q*_*s*_ with the MOST technique and the Holtslag correction led to a negative overestimation that averaged − 57 Wm^−2^ (− 40 to – 73 Wm^−2^ for the 25th–75th percentiles), almost doubling the EC measurements (see slopes in Fig. S1a,b). In contrast, MOST Q_s_ values were estimated around 0 Wm^−2^ during calm nights, when the near-surface air temperature gradient was absent, and where EC measurements were not reliably comparable since they did not pass the NaN test. The error of the parameterized Qs is significantly reduced by replacing z_0_ with the value calculated using P84 or by using the 3LM approach (Fig. 3d). The clear improvement caused by using z_0_ with P84 formula indicates that the primary source of error lies in the estimation of z_0_. Furthermore, the reduced error in the 3LM suggest a very strong gradient in the molecular and buffer layers compared with the gradient in the turbulent layer. The fact that the surface temperature modeled with the 3LM deviates temporarily (by up to 3 K) from the measured surface temperature, especially in the first half of the investigation period in the Alpine Valley, might indicate temporary decoupling. Using EC measurements of *Q*_*s*_ compared to the thermal gradient, we obtained an almost constant *C*_*s*_, with a mean of 1.9 × 10^–3^ (1.2–1.9 × 10^–3^ for the 25th–75th percentiles) throughout the period, which contrasts with the *C*_*s*_ estimated from MOST with a mean of 3.8 × 10^–3^ (3.2–4.4 × 10^–3^ for the 25th–75th percentiles). After applying the calculated *C*_*s*_ into the C-method, we obtained a closer representation of *Q*_*l*_ measurements than with the MOST method with a slight underestimation averaging -7 Wm^−2^ (5–10 Wm^−2^ for the 25th–75th percentiles) compared to the EC measurements (Fig. 3e). A similar offset is found using z_0_ calculated according to P84 or after applying the C-method to the 3LM values of Q_s_. Instead, MOST overestimated sublimation (i.e. positive latent heat fluxes) with values averaging 22 Wm^−2^ (10–35 Wm^−2^ for the 25th–75th percentiles) higher than EC measurements.

The period analysed in the Antarctic Plateau was recorded in early spring with air temperatures as low as − 40 °C (Fig. 3g). This period contains two subperiods with different weather conditions. From 3rd to 5th March, wind had a south-easterly component with speeds generally below 5 ms^−1^ (Fig. 3h). Furthermore, this period was characterized by a high near-surface thermal gradient, especially during the night, when the surface temperature dropped more markedly than the near-surface air temperature (Fig. 3g). On 5 March, wind direction shifted to the north-east and wind speeds increased over 5 ms^−1^. With north-easterly winds, air temperature increased to around − 25 °C and both the diurnal cycle and the near-surface air temperature gradient decreased markedly. The increase of the wind on 8 March might have produced blowing snow, as we see a decrease in snow height and an increase of the spikes in the EC measurements (not shown). In this case, MOST estimates closely follow the trend of the EC measurements with a mean underestimation of *Q*_*s*_ of − 5 Wm^−2^ (− 1 to − 12 Wm^−2^ for the 25th–75th percentiles) that is reduced to 0.2 Wm^−2^ using the P84 z_0_. Note as well that the agreement between MOST and EC is still good even though we expect that snow transport was happening. In this case, the difference between the 3LM and the EC measurements (Fig. 3j) suggests the presence of a shallow decoupling below the measurement height.

Both sub-periods presented slightly different *C*_*s*_, with values averaging 1.3 × 10^–3^ (1.1–1.4 × 10^–3^ for the 25th–75th percentiles) between 3 and 5 March and 2.0 × 10^–3^ (1.4–2.1 × 10^–3^ for the 25th–75th percentiles) between 5 and 9 March. While the *C*_*s*_ in the second period was well captured by the MOST estimates, it was slightly overestimated during the first period. Differences in exchange coefficients between periods were correlated with the near-surface temperature gradient, leading to different magnitudes of *Q*_*l*_ calculated with the C-method in both subperiods. *Q*_*l*_ values with C-method averaged 0.6 Wm^−2^ (compared with − 0.2 Wm^−2^ measured using EC) before 5 March and 4.2 Wm^−2^ (compared with 2.3 Wm^−2^ measured using EC) afterwards. In contrast, MOST estimations averaged 0.8 and 4.3 Wm^−2^ (0.6 and 3.4 Wm^−2^ with P84 z_0_) in the first and second subperiods, respectively, and presented overall a similar representation than the C-method (Fig. 3e, f). The two peaks observed with this method occur when vertical temperature difference and thus the denominator in Eq. 8 approach zero, exhibiting the main limitations of the C-method. Note, however that with the low temperature absolute values the latent heat fluxes remain very small.

### Statistics and Sensitivity Analysis

The statistical evaluation of both datasets indicates that the C-method is comparable to, or better than, the MOST method in reproducing latent heat fluxes measured with the EC method over snow. Overall, the C-method yields a median error of 7.5 Wm^−2^ (17%) and 2.0 Wm^−2^ (7%) in the Alpine Valley and Antarctic Plateau, respectively, which agrees with the error margin shown by Liu and Foken (2001) for other surface types. In contrast, the MOST method with Holtslag stability correction showed much higher errors with means of 23.9 Wm^−2^ (65%) and 2.2 Wm^−2^ (14%), respectively. Results of the mean absolute error (MAE) and bias are shown in Tables 2 and 3. We tested different MOST stability corrections and found that MAE values ranged from 6.1 to 30.1 Wm^−2^ in the Alpine Valley and 1.8–4.3 Wm^−2^ in the Antarctic Plateau. Only the Schlögl multivariate correction in the Alpine Valley dataset performed better than the C-method (MAE = 6.1 Wm^−2^ with BIAS = 1.9 Wm^−2^). However, the same method struggled to accurately represent the latent heat flux in the Antarctic Plateau compared to the other methods (MAE = 4.3 Wm^−2^), making it the stability correction with the worst performance at that site. In contrast, all the other stability corrections performed similar or slightly worse than the C-method, with Stearns being the best performer over the Antarctic Plateau. We also conducted a sensitivity analysis with respect to the chosen value of roughness length. Table 3 shows that changes in roughness length led to significant adjustments in MOST performance, with higher accuracy using shorter roughness lengths, showing the overestimation of this parameter. In general, MOST estimations are more sensitive to stability corrections and roughness length in the Alpine Valley, where the fluxes are higher, than in the Antarctic Plateau.

**Table 2:** Q_s_ and Q_l_ BIAS and MAE (W m^−2^) for the C-method and MOST with different stability corrections compared with EC measurements

	C-method	Stability correction for MOST						
		Richardson	Neutral	LogLinear	Holtslag	Stearns	Schlögl Uni	Schlögl Multi
*Alpine Valley*								
Q_s_ MAE	–	63.7	72.2	58.6	**58.4**	41.7	63.4	15.6
Q_s_ BIAS	–	− 63.4	− 72.2	− 58.6	**− 58.4**	− 41.6	− 63.4	− 15.3
Q_l_ MAE	7.5	27.1	30.1	23.8	**23.9**	15.9	25.9	6.1
Q_l_ BIAS	− 7.2	24.7	28.0	21.8	**22.0**	13.8	23.9	1.9
*Antarctic Plateau*								
Q_s_ MAE	–	8.9	9.4	7.2	**7.2**	4.3	8.1	9.0
Q_s_ BIAS	–	− 7.9	− 8.5	− 6.2	**− 6.2**	− 1.9	− 7.1	− 5.8
Q_l_ MAE	2.0	2.3	2.4	2.2	**2.2**	1.8	2.3	4.3
Q_l_ BIAS	1.7	2.3	2.4	2.2	**2.2**	1.8	2.3	4.3

The bold results are used as a reference

**Table 3:** Q_s_ and Q_l_ BIAS and MAE (W m^−2^) for the C-method and MOST with different fractions of the calculated roughness length compared with EC measurements

	C-method	Fraction of the calculated roughness length for MOST				
		1/3	2/3	1	4/3	5/3
*Alpine Valley*						
Q_s_ MAE	–	31.0	46.5	**56.6**	67.0	75.7
Q_s_ BIAS	–	− 31.0	− 46.5	**−56.6**	− 67.0	− 75.7
Q_l_ MAE	7.6	9.1	15.6	**21.0**	24.4	28.0
Q_l_ BIAS	− 7.3	7.0	13.5	**18.7**	22.2	25.8
*Antarctic Plateau*						
Q_s_ MAE	–	3.9	5.5	**7.5**	9.2	11.1
Q_s_ BIAS	–	0.2	− 3.7	**−6.6**	− 8.6	− 10.7
Q_l_ MAE	1.8	1.8	2.2	**2.5**	2.8	3.0
Q_l_ BIAS	1.7	1.5	2.0	**2.3**	2.5	2.8

The bold results are used as a reference

**Fig. 4:**
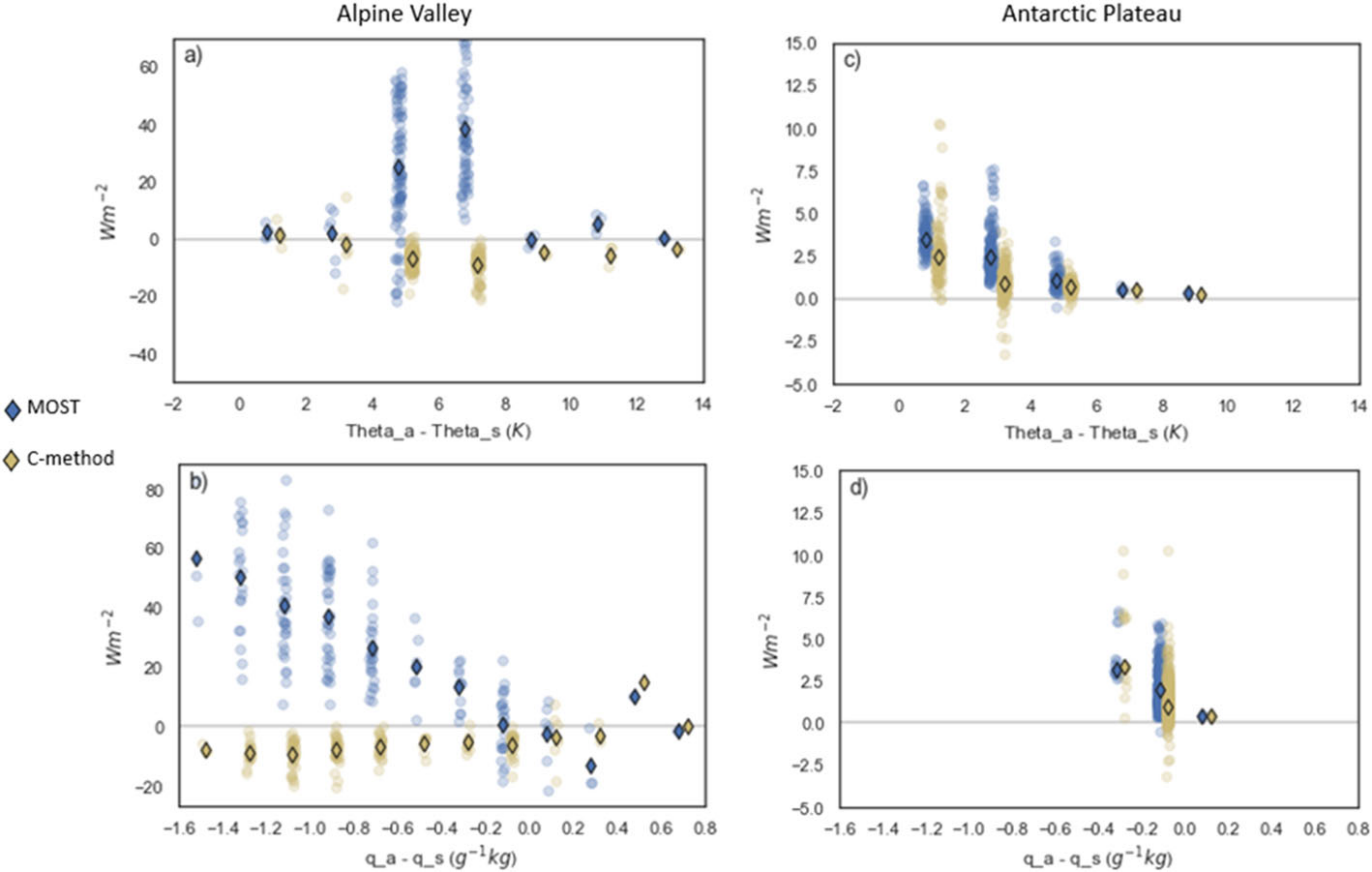
Difference between Ql using the MOST method (blue) and the C-method (yellow) with respect to the EC measurements, as function of the vertical potential temperature difference (**a**, **c**) and the vertical specific humidity difference (**b**, **d**) for the Alpine Valley (**a**, **b**) and the Antarctic plateau (**c**, **d**) datasets. Outlined diamonds represent the median of the values. MOST calculations shown in this figure use Holtslag stability correction and the estimated roughness length

Figure 4a, c compares the absolute differences of the C-method and the MOST method with Holtslag stability correction with respect to the potential temperature difference between the near-surface air and the surface (see the other corrections in Fig. S2). The MOST method shows a good accuracy and low scatter at potential temperature differences over 8 K and below 4 K in the Alpine valley. Maximum errors in the Alpine Valley occur in the range between 4 and 8 K potential temperature difference, where most values are available, and where MOST overestimates Q_l_ by more than 20 Wm^−2^ on average. In this range, the C-method improves on the MOST method with all corrections except for the Schlögl multivariate correction (see Fig. S3). Instead, in Antarctica, performance is similar for both the C-method and the MOST method for any stability correction, with a little improvement with C-method. Improved performance is obtained compared to the Schlögl multivariate correction at low vertical potential temperature differences (Fig. S3).

The deviation of both methods with respect to the difference in specific humidity is shown in Fig. 4b, d. In the Alpine Valley dataset, the C-method generally underestimates *Q*_*l*_ by approximately 10 Wm^−2^ for any negative moisture gradient. Instead, the MOST method overestimates *Q*_*l*_, increasing with the specific humidity difference, for any correction except for the Schlögl multivariate (Fig. S4). Both methods perform similar for positive moisture gradients. In Antarctica, due to low air humidity capacity, the moisture difference never exceeded 0.4 g kg^−1^. Although median deviations are low, there is a large scatter in relative differences, especially for small humidity differences. In this case, the C-method performs similarly as MOST, or better when compared to the Schlögl multivariate correction (Fig. S5).

Application in High Mountain Asia.

We applied the methodology to a newly installed station in the Pamir Mountains with high-frequency measurements (20 Hz) of wind velocity components. Because of the station's remote location, which allows for maintenance and data collection only once a year, a high-frequency water vapour flux device was not installed. Neutral stability measurements indicate a z_0_ of 0.17 m during the snow season due to the steep terrain with rocks surrounding the station and the P84 method reduces it to 0.05 m.

**Fig. 5:**
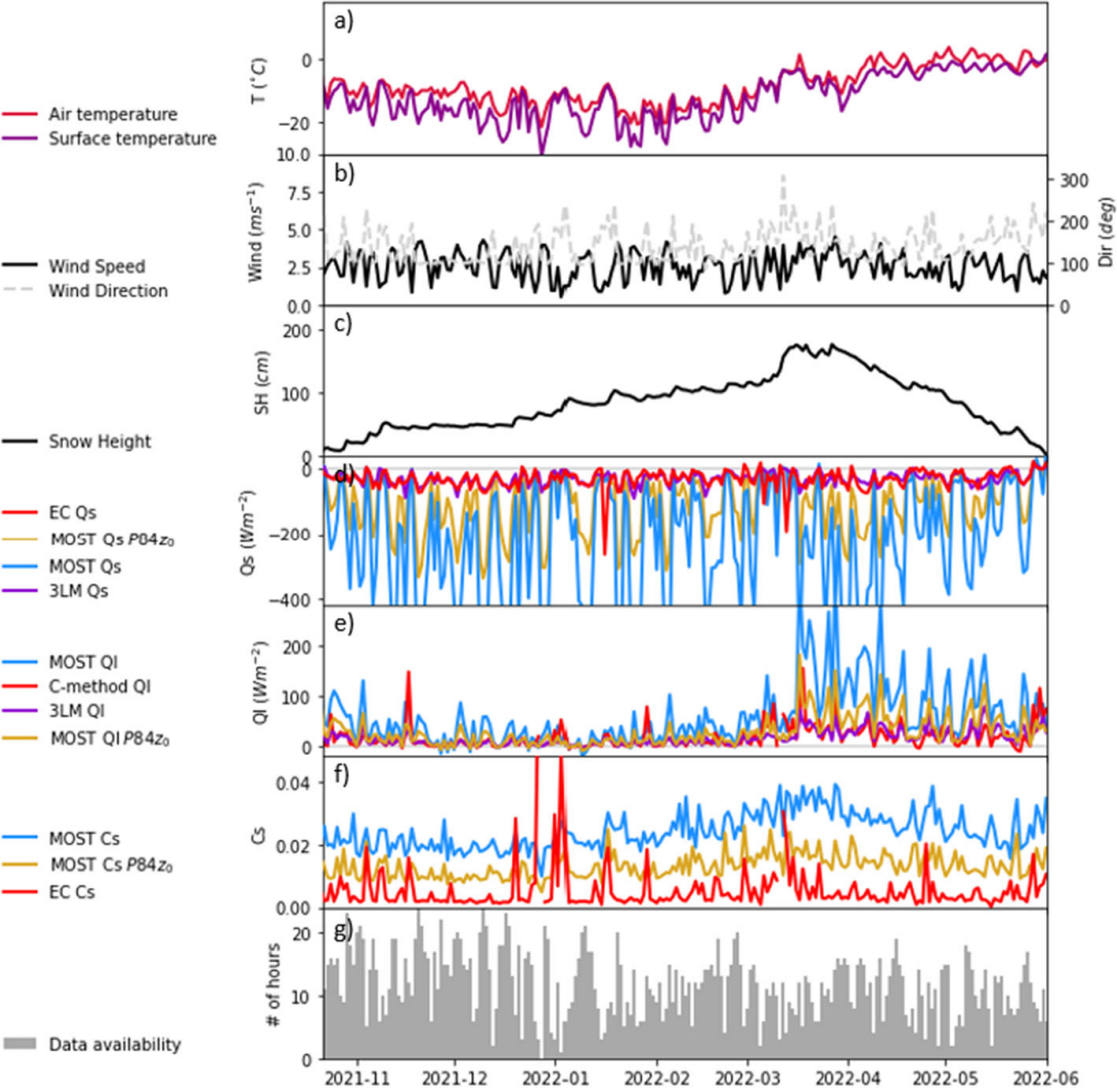
As Fig. 2 but for the snow season in the Pamir mountains. Values correspond to daily averages of hourly values. We added the data availability per day in (g)

Figure 5 shows the daily mean values of the 2021–2022 snow season, which lasted from 21 October 2021 to 01 June 2022, and had a snowpack height up to 2 m. Mean near-surface air temperatures ranged from 0 to − 20 °C. The C-method applied to the valid data yielded a mean *Q*_*l*_ of 17.3 Wm^−2^, significantly below the 59.6 Wm^−2^ calculated using the MOST method. Most of the higher estimations of the MOST method with respect to the presented one occurred mainly from mid-March during the melt season. If we use the MOST method with P84 z_0_, we obtain a value of *Q*_*l*_ of 29.2 Wm^−2^.

Using the C-method, the estimated average sublimation rate during the snow season was 0.67 mm day^−1^, which is in the range of other high mountain estimates (e.g. Reba et al. 2012; Stigter et al. 2018). This estimate increases to values of 2.32 mm day^−1^ using the MOST method and a z_0_ of 0.17 m, which seems high compared with other mountain estimates. With a z_0_ value of 0.05 m calculated using P84, the value of 1.14 mm day^−1^ might be considered still high (notice that Cs in Fig. 5f is still overestimated with this roughness length). Finally, the 3LM provides an estimate of 0.64 mm day^−1^, which is comparable to the C-method indicating that it might be a good alternative in absence of high-frequency measurements. The clear advantage of the C-method and 3LM applied to the Pamir station is that they avoid arbitrarily choosing a stability correction method and the roughness length, which significantly affects our results.

## Discussion and Concluding Remarks

Precise values for sensible and latent heat fluxes over snow are crucial for understanding the surface energy- and mass- balances, as well as the thermal and hydrological properties of the snowpack. However, several challenges exist due to the influence of the roughness length, stability of the atmospheric boundary layer and potential decoupling. The roughness length is a key factor in determining the transfer of momentum, heat and moisture between the surface and the atmosphere. Snow-covered surfaces are characterized by small roughness lengths but with very variable scales. They are not only dependent on the characteristic roughness length of the snow itself, which is in the order of tenths of millimeters (Clifton et al. 2008; Gromke et al. 2011), but also on the macroscopic objects and obstacles in the surroundings (Clifton et al. 2006; Stössel et al. 2010). We observed that different methods used to estimate the roughness length yielded to very different z_0_ values and *Q*_*l*_ estimates from MOST are highly sensitive to the chosen roughness lengths, which are in turn very sensitive to the uncertainty of the wind measurements or the calculation method. We show that the P84 formulation to obtain z_0_ has a good performance over snow surfaces. However, this formula needs high-frequency measurements of wind speed which are not always available and it seems not to have the best performance in the Pamir dataset. A good alternative is using flux derivations that do not need z0, such as the 3LM. However, it’s applicability in a wider context needs to be investigated; we note here that it contains empirically derived parameters.

**Fig. 6:**
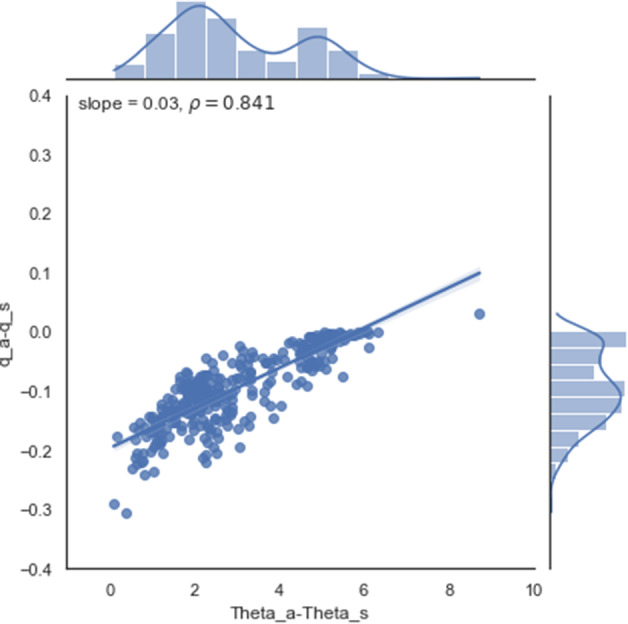
Similarity between the vertical gradients of temperature and specific humidity

Stable conditions over the snow can also limit the accuracy of flux estimates. Empirical functions developed to correct for stability are site and regime specific, which makes them less reliable (Grachev et al. 2007; Srivastava et al. 2020). The stability formulations developed for MOST work better in a weakly stable boundary layer. However, the accuracy depends on time and space and we could not find a single best correction. A paradigmatic example presented here is the Schlögl Multivariate stability correction, which is the best-performing correction in the Alpine Valley and the worst in Antarctica. The C-method became especially useful in regions with a wide stability regime since it does not depend on the (almost) arbitrary selection of an empirical function. The presence of a strongly stable stratification in the atmospheric boundary layer, which is common over snow and at night-time limits the vertical mixing of heat and moisture between the surface and the atmosphere and leads to intermittent turbulence (Grachev et al. 2005, 2013; Cullen et al. 2007). This can be seen in the 3LM, which corrects MOST calculations in all our datasets. Our results suggest that z_0_ is our primary source of error over snow and ice. In absence of reliable measurements of z_0_, the 3LM is preferable to estimations using MOST. However, strongly stable stratification also affects the EC measurements, since it causes non-turbulent motions and intermittent turbulence to violate the steady-state assumption. In that case, the turbulent flux measured at some height does not correspond to the exchange at the surface. Moreover, such conditions can lead to vertical decoupling of atmospheric low levels below the instrumentation, creating counter-gradient fluxes, for which the similarity theory assumptions clearly do not work (Mott et al. 2016; Foken 2023). Nevertheless, the C-method seems to be robust in the different cases presented. An interesting question arises regarding why the C-method remains effective even during periods of strong surface gradient, decoupling or drifting snow. We suggest that this occurs because both the temperature and water vapour are transported in the same way throughout the surface layer, including the buffer and the molecular sublayers. In addition, over snow and ice, surface temperature changes are often closely tied to corresponding vapour changes because surface conditions stay close to saturation. Thus, we can speculate that scalar profiles for strong surface gradients and decoupling may be similar, which is not a necessary requirement of the C-method to work but could help to limit potentially differing behaviour of the scalar fluxes across buffer and molecular sublayers. As an example, Fig. 6 illustrates this similarity on the Antarctic plateau.

The method presented here to calculate the latent heat flux, which has previously been presented as the modified Bowen ratio method, does not need any assumption or arbitrary choice of either the roughness length or stability corrections and reliably approximates the latent heat flux calculated by the EC method. Still, it needs high-frequency measurements of the three components of the wind and sonic temperature, low-frequency air and surface temperature, and the proper postprocessing of the sensible heat flux measurements. If those are available, it provides a method to extend the latent heat flux estimations at such sites without using MOST methods with more arbitrary choices. In absence of high-frequency wind and temperature measurements, we recommend to use the 3LM-based sensible heat flux as input for the C method to obtain good seasonal estimates of sublimation.

### Supplementary Information

Supplementary file 1

## Data Availability

The datasets and codes to reproduce this research are available at Envidat published as González-Herrero, S., Lehning, M. (2023). Latent Heat Flux over Snow. *EnviDat.* https://www.doi.org/10.16904/envidat.463.
