# Supplementary material for: Using the Sensible Heat Flux Eddy Covariance-Based Exchange Coefficient to Calculate Latent Heat Flux from Moisture Mean Gradients Over Snow
Source: Boundary Layer Meteorol. 2024 May 4;190(5):24. doi: 10.1007/s10546-024-00864-y (PMC11068579; doi:10.1007/s10546-024-00864-y)
Supplement: Supplementary file 1 [file 10546_2024_864_MOESM1_ESM.docx]

Target journal: Boundary-Layer Meteorology

# Using the sensible heat flux eddy covariance based exchange coefficient to calculate latent heat from moisture mean gradients over snow

Sergi González-Herrero^1^, Armin Sigmund^2^, Michael Haugeneder^1,2^, Océane Hames^1,2^, Hendrik Huwald^2^, Joel Fiddes^1^, Michael Lehning^1,2^

^1^WSL Institute for Snow and Avalanche Research (SLF), Davos, Switzerland

^2^School of Architecture, Civil and Environmental Engineering, Ecole Polytechnique Fédérale de Lausanne, Lausanne, Switzerland


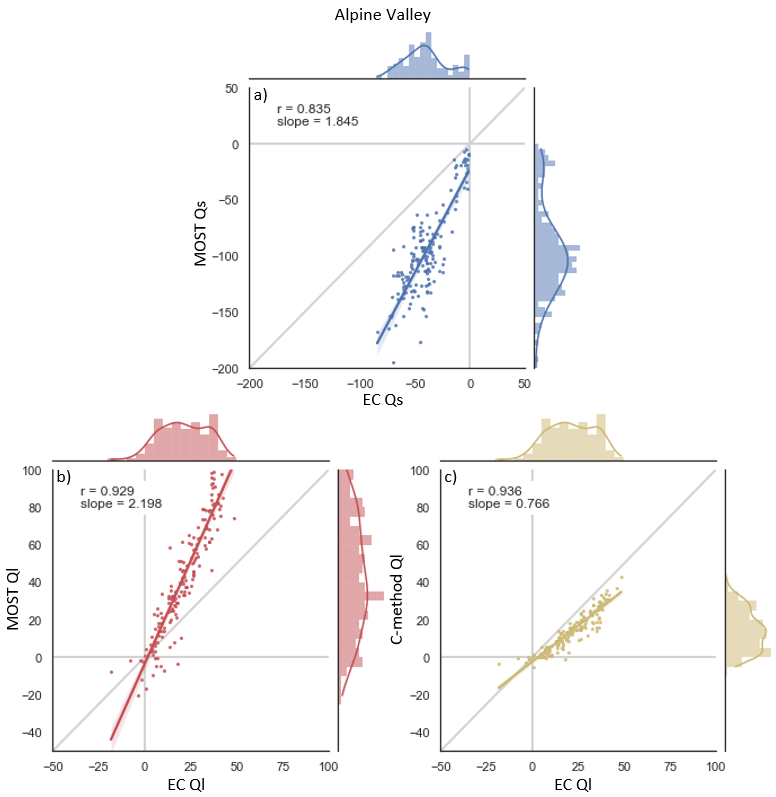

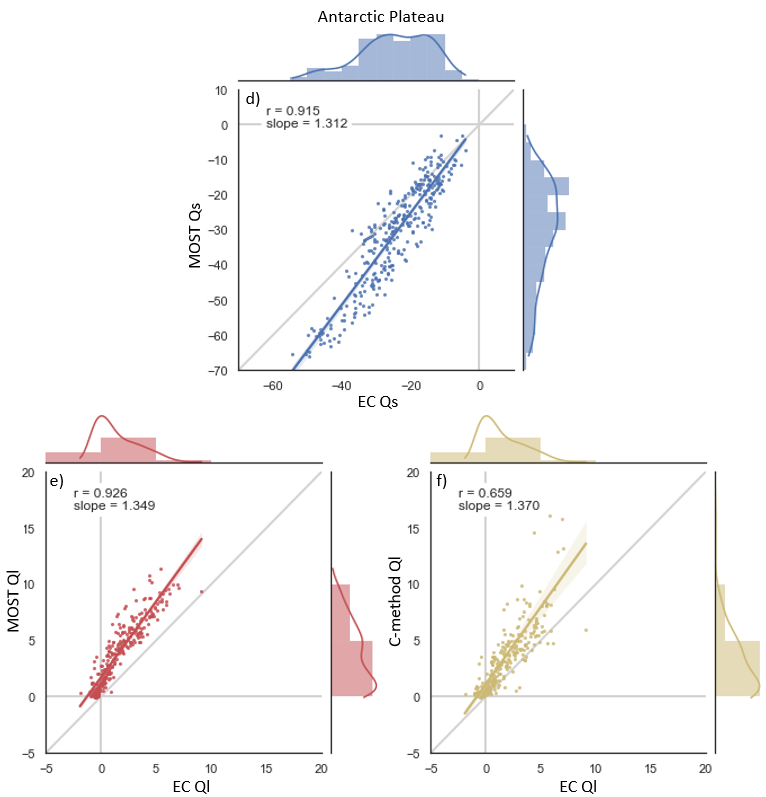


**Figure S1.** Scatter plot and regression fit of MOST and EC Qs (a,d), MOST and EC Ql (b,e) and the new method and EC Ql (c,f) for the Alpine Valley (a,b,c) and the Antarctic plateau (d,e,f) datasets. MOST calculations shown in this figure use Holtslag stability correction and the estimated roughness length.


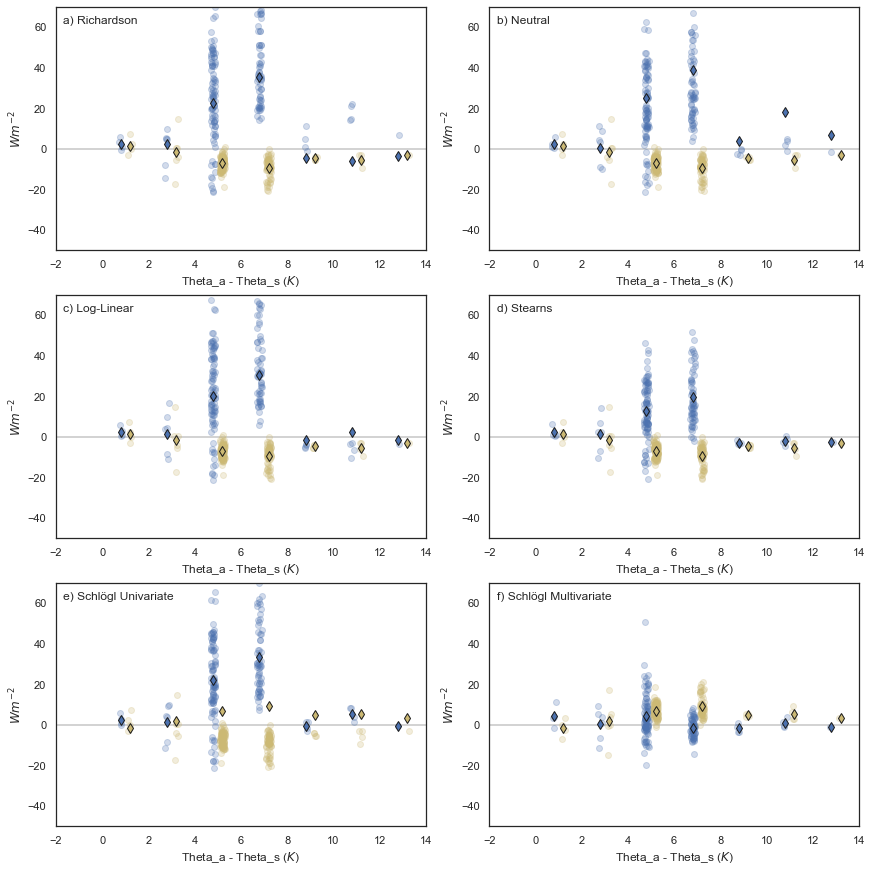


**Figure S2.** As in Figure 4a but for different stability corrections.


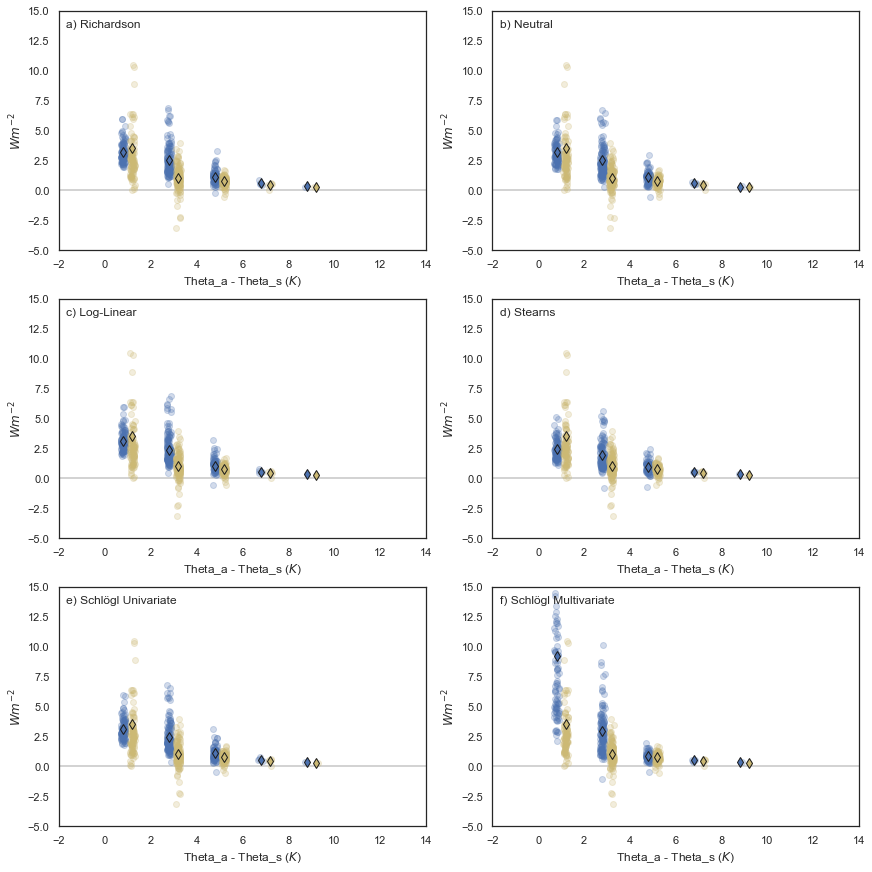


**Figure S3.** As in Figure 4c but for different stability corrections.


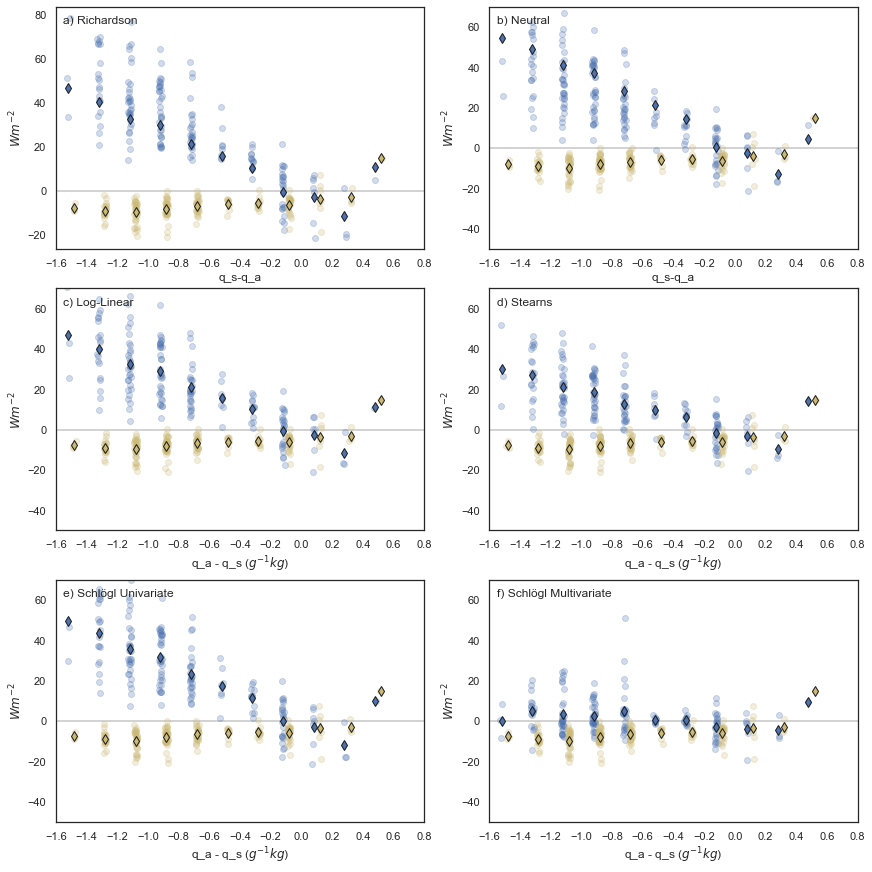


**Figure S4.** As in Figure 4b but for different stability corrections.


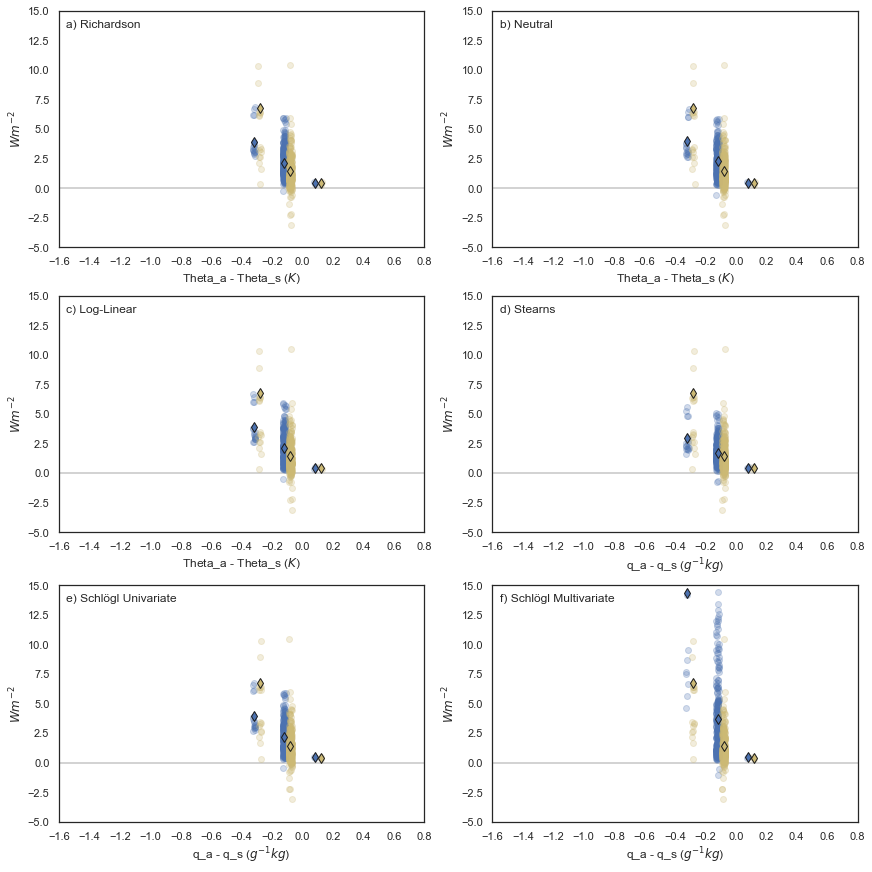


**Figure S5.** As in Figure 4d but for different stability corrections.
